# A Weather-Adaptive Convolutional Neural Network Framework for Better License Plate Detection

**DOI:** 10.3390/s24237841

**Published:** 2024-12-08

**Authors:** Utsha Saha, Binita Saha, Md Ashique Imran

**Affiliations:** 1 Department of Computer Science, North Dakota State University, Fargo, ND 58105, USA; binita.saha@ndsu.edu; 2 Applied Computer Science, University of Winnipeg, Winnipeg, MB R3B2E9, Canada; imran-m91@webmail.uwinnipeg.ca

**Keywords:** intelligent transport systems (ITS), automatic license plate recognition (ALPR), YOLOv10, convolutional neural networks (CNN), object detection, traffic monitoring

## Abstract

Automatic License Plate Recognition (ALPR) systems are essential for Intelligent Transport Systems (ITS), effective transportation management, security, law enforcement, etc. However, the performance of ALPR systems can be significantly affected by environmental conditions such as heavy rain, fog, and pollution. This paper introduces a weather-adaptive Convolutional Neural Network (CNN) framework that leverages the YOLOv10 model that is designed to enhance license plate detection in adverse weather conditions. By incorporating weather-specific data augmentation techniques, our framework improves the robustness of ALPR systems under diverse environmental scenarios. We evaluate the effectiveness of this approach using metrics such as precision, recall, F1, mAP50, and mAP50-95 score across various model configurations and augmentation strategies. The results demonstrate a significant improvement in overall detection performance, particularly in challenging weather conditions. This study provides a promising solution for deploying resilient ALPR systems in regions with similar environmental complexities.

## 1. Introduction

Automatic License Plate Recognition (ALPR) has become a cornerstone technology in Intelligent Transport Systems (ITS), offering advanced capabilities for vehicle identification and tracking [1]. By recognizing and recording the unique characters on license plates, ALPR systems not only identify vehicles but also differentiate between each one [2]. These systems are essential in transportation management, law enforcement, and security, playing critical roles in tasks such as traffic monitoring, toll collection, and criminal investigations, particularly in regions with high traffic density and diverse vehicle types [3,4].

**Figure 1:**
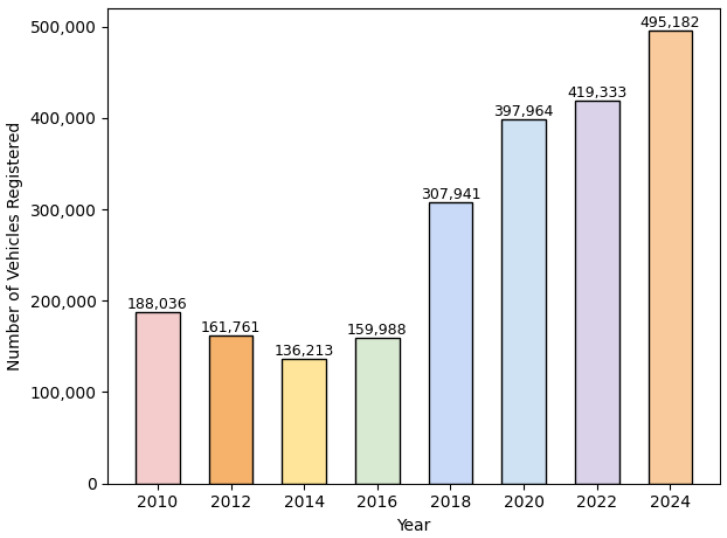
Motor vehicle registration in Bangladesh (2011–2023) [6].

Bangladesh, the focus of this study, is a country facing significant traffic congestion and a rapidly growing vehicle population characterized by a diverse road network and different license plate designs [5]. According to the Bangladesh Road Transport Authority (BRTA), there has been substantial growth in the number of vehicles over the past decade, underscoring the urgent need for effective monitoring systems to handle increasing traffic demands (see Figure 1) [6].

Developing ALPR systems in Bangladesh is particularly challenging due to environmental factors such as heavy monsoon rains, dense winter fog, and high levels of dust and pollution. These weather conditions can obscure license plates, reduce visibility, and complicate detection and recognition. The frequent occurrence of extreme weather requires a robust and adaptable system, as traditional ALPR methods often fail to maintain good performance under these conditions.

In our previous study [7], we developed an ALPR for the Bangladesh system using YOLOv8. However, its performance declined under adverse weather conditions such as heavy rain and fog, resulting in lower detection performance (the authors use the term “performance” to refer to a combination of precision, recall, F1 score, mAP50, and mAP50-95). These limitations highlight the need for a more robust model to address such challenges.

To address this issue, we propose a weather-adaptive convolutional neural network (CNN) framework that leverages augmentation of weather-specific data with YOLOv10 [8] to enhance detection under various environmental conditions. We evaluate the proposed system using precision, recall, and mean Average Precision (mAP), comparing performance both with and without the inclusion of weather augmentation.

This paper is organized as follows: Section 2 reviews the background study and related work, Section 3 describes the dataset and data augmentation techniques, Section 4 outlines the proposed framework and experimental setup, and Section 5 presents the results. Finally, Section 6 concludes this paper, highlighting our contributions and future research directions in ALPR systems.

## 2. Background Study

Automatic License Plate Recognition (ALPR) systems have become integral components in modern security, surveillance, and transportation infrastructures. They facilitate efficient vehicle identification, which is essential for various applications such as law enforcement, traffic management, toll collection, and parking control [3,4]. By automatically capturing and recognizing license plate information, ALPR systems contribute to enhancing road safety, enforcing traffic regulations, and streamlining transportation operations.

### 2.1. Importance of License Plate Detection

License plate detection is the foundational step in ALPR systems, where the system locates the license plate region within an image or video frame. Accurate detection is critical because any error at this stage can propagate through the subsequent character recognition process, leading to incorrect vehicle identification [9]. Efficient license plate detection enables real-time monitoring and control, which is particularly important in high-traffic areas and security-sensitive zones [10].

### 2.2. Challenges in Existing Methods

Despite significant advancements, existing license plate detection methods face challenges that affect their accuracy and reliability. Common issues include:

- Varying lighting conditions: Changes in illumination, shadows, and glare can obscure license plates, making detection difficult [11,12].
- Occlusions: Partial obstruction of license plates by objects such as dirt, frames, or other vehicles hinders detection algorithms [13].
- Weather-related problems: Adverse weather conditions like rain, snow, fog, and haze introduce noise and distortions in images, reducing the clarity of license plates [10].
- Variations in license plate designs: Differences in plate formats, fonts, and sizes across regions pose additional challenges for detection models [14].

These challenges require the development of more robust detection methods that are capable of handling diverse conditions.

### 2.3. Recent Advancements in Deep Learning for Detection

Deep learning, particularly Convolutional Neural Networks (CNNs), has revolutionized the field of computer vision and has been widely applied to license plate detection and recognition [15]. CNN-based models, such as those in the YOLO (You Only Look Once) family, have demonstrated high accuracy and real-time performance in object detection tasks [3,16]. For instance, YOLOv5 and YOLOv8 have been employed for license plate detection due to their efficiency and effectiveness [12,16].

These models excel in learning hierarchical features from data, allowing them to handle complex backgrounds and varying plate designs [11]. Additionally, techniques like Generative Adversarial Networks (GANs) have been used to enhance image resolution, improving detection in low-quality images [10].

Recent works have further expanded the applications of deep learning in this domain. IoT-based license plate recognition systems employing single-board computer technology have demonstrated practical, cost-effective implementations, focusing on factors like camera angle, object velocity, and distance to optimize real-time performance [17]. Transfer learning with YOLOv3 and VGG16 has been applied to develop high-precision systems for Jordanian license plates, achieving mean average precision (mAP) values close to 99.9% [18]. A deformable convolution-based YOLOv8 network has also been proposed to enhance detection accuracy, surpassing traditional YOLO models by a significant margin [19]. Furthermore, an enhanced YOLOv8-based system tailored for Qatar’s unique license plate and environmental conditions has shown promising results in real-time automatic number plate recognition [20].

### 2.4. Motivation for a Weather-Adaptive Approach

While deep learning models have improved license plate detection, their performance often degrades under adverse weather conditions. Weather phenomena such as rain, fog, and snow can obscure license plates and alter image characteristics, leading to reduced detection accuracy [10]. Therefore, there is a pressing need for models that can adapt to different weather conditions and maintain high performance.

A weather-adaptive approach aims to enhance the robustness of license plate detection models by incorporating mechanisms to handle weather-induced variations in images. This could involve training models on weather-augmented datasets or integrating weather recognition modules to adjust processing accordingly [11].

### 2.5. Gaps in Current Research

Despite the progress in applying deep learning to license plate detection, gaps remain in addressing the challenges posed by varying weather conditions. Many existing models are trained on datasets that lack sufficient diversity in weather scenarios, limiting their generalization capabilities [14]. Furthermore, few studies have focused specifically on developing weather-adaptive frameworks for license plate detection.

Current methods often do not account for the dynamic nature of environmental conditions, resulting in performance degradation when deployed in real-world settings [10]. This highlights the necessity for research into adaptive models that can maintain performance across different weather conditions.

### 2.6. Our Contribution

In light of these challenges, we propose a weather-adaptive convolutional neural network framework designed to improve license plate detection under diverse environmental conditions. Our approach aims to enhance overall detection accuracy and robustness by incorporating weather adaptation mechanisms, addressing the limitations of current methods.

## 3. Materials

The dataset used in this research, as described by Hossain et al. [21], was specifically developed for Automatic License Plate Recognition (ALPR) of Bangladeshi vehicles. It encompasses a broad spectrum of conditions, angles, and settings, representing license plates from 12 vehicle types across four major cities in Bangladesh: Dhaka, Khulna, Chattogram, and Jashore. This extensive dataset includes trading vehicles cited by Nooruddin et al. [22], private vehicles referenced by Rahman [23], and additional images collected by the authors. In our earlier work [7], the dataset’s sections were combined into a unified collection, which serves as the foundational dataset for this study.

### 3.1. Structure of Bangladeshi License Plates

**Figure 2:**
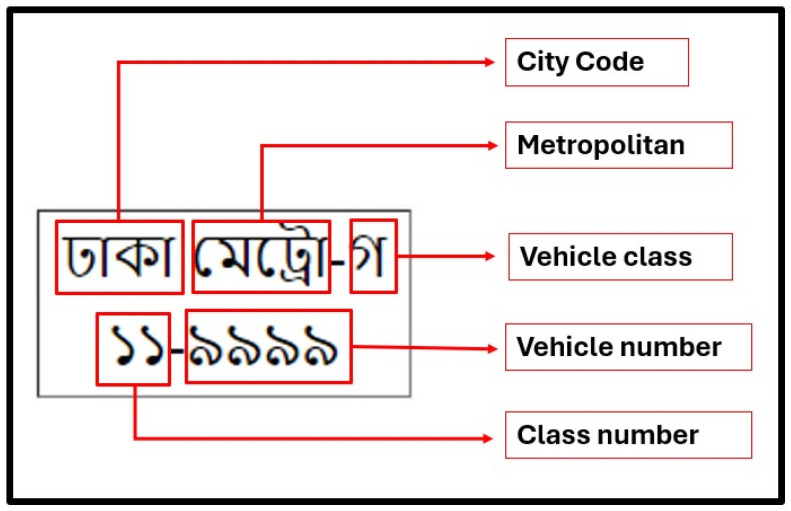
Structure of Bangladeshi license plate.

Bangladeshi license plates have a unique structure that combines both Bangla and English characters, typically containing a mix of numeric and alphabetic components. The design varies depending on the type of vehicle, such as private cars, commercial vehicles, or government-owned vehicles. The plates usually feature a top section with the regional code in Bangla, followed by a sequence of numbers in both Bangla and English. Figure 2 provides an illustration of the typical structure of a Bangladeshi license plate [24].

This structure poses additional challenges for ALPR systems due to the presence of non-standardized fonts, varying character sizes, and the dual-language format. Furthermore, environmental factors such as dirt, wear-and-tear, and different plate colors add complexity to detection and recognition tasks.

### 3.2. Data Augmentation

To enhance model performance and robustness, a combination of general and weather-specific augmentations was applied to simulate diverse real-world scenarios [25]. These techniques ensured reliable object detection under varying conditions, including challenging weather, non-standard orientations, and diverse lighting.

#### 3.2.1. General Augmentations

General augmentations, including flipping, scaling, rotation (±15° and ±90°), and color jittering, improved the model’s resilience to variations in angles, scales, and lighting [26,27]. Brightness, contrast, and saturation adjustments simulated conditions such as shadows, underexposure, and overexposure, forming a robust foundation for standard scenarios.

#### 3.2.2. Weather-Specific Augmentations

Weather-specific augmentations replicated adverse conditions to further enhance robustness [28,29,30,31]. Synthetic rain with varying density was used to simulate rainfall, while fog and haze effects mimicked low-visibility scenarios like smog or early mornings. Brightness and contrast adjustments accounted for lighting extremes, and noise injection (Gaussian and salt-and-pepper) simulated sensor noise and pixelation in corrupted images. The weather-specific augmentations were implemented using the Python library imgaug [32].

#### 3.2.3. Systematic Augmentation Process

The augmentation process systematically combined general and weather-specific transformations. For each input image, a random subset of augmentations was applied to replicate real-world variability. Parameters such as rain density, fog intensity, and brightness scaling were randomized to ensure diversity. A subset of augmented images was visually inspected to confirm realism.

**Figure 3:**
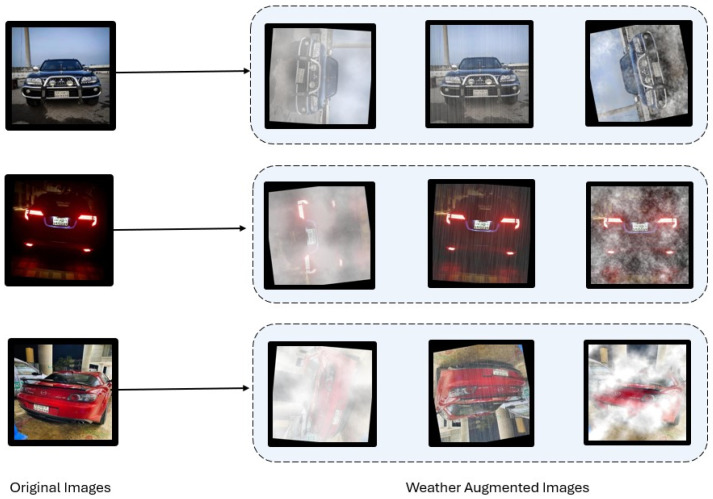
Different augmentations applied to the dataset.

Figure 3 illustrates key weather-specific augmentations combined with other augmentation techniques. We started with a dataset of 28,718 images, and after applying a thoughtful combination of general and weather-specific augmentations, it grew to 125,914 images. This process not only added variety but also ensured the model is better prepared to handle the complexities of real-world scenarios.

Table 1 summarizes the various augmentation techniques used in the training process. This diverse set of augmentations ensured that the YOLOv10 model was trained on a rich dataset, enabling accurate object detection even in adverse conditions and unusual orientations.

### 3.3. Models Used

In this study, we tested different YOLOv10 models due to their versatility and performance across various configurations, each tailored to meet specific deployment and application needs. YOLOv10 offers several configurations, each tailored to specific deployment and application needs. The performance metrics of YOLOv10 variants are summarized in Table 2.

For example, YOLOv10-S is 1.8 times faster than RT-DETR-R18 while maintaining a similar Average Precision (AP) on the COCO dataset. Additionally, YOLOv10-B demonstrates 46% lower latency and 25% fewer parameters compared to YOLOv9-C, achieving comparable performance [33]. The Nano variant (YOLOv10-N) is optimized for extremely resource-constrained environments, such as microcontrollers and low-power devices. The small version (YOLOv10-S) balances efficiency and accuracy, making it well-suited for mobile and embedded systems. For general-purpose applications, the medium variant (YOLOv10-M) offers a versatile option with improved accuracy while maintaining reasonable resource demands. The balanced model (YOLOv10-B) increases model width to achieve greater precision, which is ideal for scenarios where moderate computational resources are available. The large version (YOLOv10-L) is designed for tasks requiring high accuracy, trading off increased computational requirements, and it is suitable for edge servers or more capable platforms. Lastly, the extra-large variant (YOLOv10-X) provides maximum accuracy and performance, making it the best choice for applications demanding exceptional precision and abundant computational resources.

## 4. Methods

### 4.1. Training Process

We conducted the training process for the YOLOv10 models using the configuration outlined in Table 3. To ensure robust model performance under various environmental conditions, we applied the data augmentation techniques described in Table 1.

During the training phase, we initialized the model with pre-trained weights from the COCO dataset to accelerate convergence. We employed a cosine learning rate scheduler to dynamically adjust the learning rate, starting at 0.01 and gradually reducing it over the course of 50 epochs.

**Figure 4:**
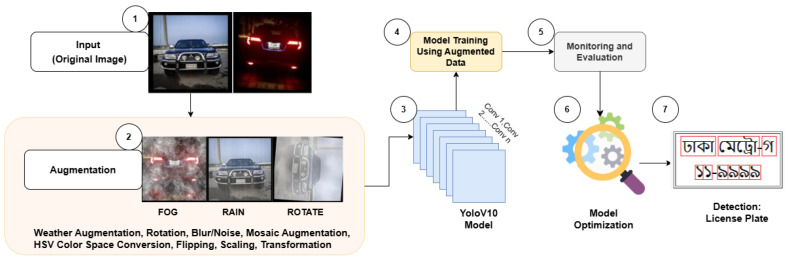
Overview of the YOLOv10 training process, including data augmentation, model training, evaluation, and license plate detection.

The entire training process is illustrated in Figure 4, which provides a detailed overview of the training workflow. This diagram highlights the key steps, from input image pre-processing to final license plate detection, showcasing how each stage contributes to the overall process.

### 4.2. Evaluation Metrics

Throughout training, several key metrics were monitored to evaluate the model’s performance:

- Precision: The ratio of correctly predicted positive observations to total predicted positives, defined as follows:
  (1) $$\mathrm{Precision}=\frac{\mathrm{True}\;\mathrm{Positives}\;\left(\mathrm{TP}\right)}{\mathrm{True}\;\mathrm{Positives}\;\left(\mathrm{TP}\right)+\mathrm{False}\;\mathrm{Positives}\;\left(\mathrm{FP}\right)}.$$
- Recall: The ratio of correctly predicted positive observations to all observations in the actual class, expressed as follows:
  (2) $$\mathrm{Recall}=\frac{\mathrm{True}\;\mathrm{Positives}\;\left(\mathrm{TP}\right)}{\mathrm{True}\;\mathrm{Positives}\;\left(\mathrm{TP}\right)+\mathrm{False}\;\mathrm{Negatives}\;\left(\mathrm{FN}\right)}.$$
- F1 score: The harmonic mean of precision and recall, offering a balanced performance metric:
  (3) $$F1\;\mathrm{Score}=2\;\cdot \;\frac{\mathrm{Precision}\;\cdot \;\mathrm{Recall}}{\mathrm{Precision}+\mathrm{Recall}}.$$
- Mean Average Precision (mAP): The average precision across all classes and object sizes, given as follows:
  (4) $$\mathrm{mAP}=\frac{1}{N}\sum_{i=1}^{N}{\mathrm{AP}}_{i},$$
  where *N* is the number of classes, and ${\mathrm{AP}}_{i}$ is the average precision for the *i*-th class.
- mAP50: Mean average precision at an Intersection over Union (IoU) threshold of 0.50, defined as follows:
  (5) $$\mathrm{mAP}50=\frac{1}{N}\sum_{i=1}^{N}{\mathrm{AP}}_{i}\;\mathrm{at}\;\mathrm{IoU}\;\geq 0.50.$$
- mAP50-95: Mean average precision averaged across multiple IoU thresholds (0.50 to 0.95 in increments of 0.05), calculated as follows:
  (6) $$\mathrm{mAP}50-95=\frac{1}{10N}\sum_{i=1}^{N}\sum_{j=0}^{9}{\mathrm{AP}}_{i}\;\mathrm{at}\;\mathrm{IoU}\;\geq 0.50+0.05j.$$

These metrics were calculated after each epoch to track the model’s performance across the training and validation sets.

### 4.3. Detection Process

After training, we evaluated the YOLOv10 model on the test set, utilizing three detection scales (P3, P4, and P5) to identify license plates of varying sizes and orientations. Non-maximum suppression (NMS) was applied to filter overlapping bounding boxes and ensure precise detections [34].

**Figure 5:**
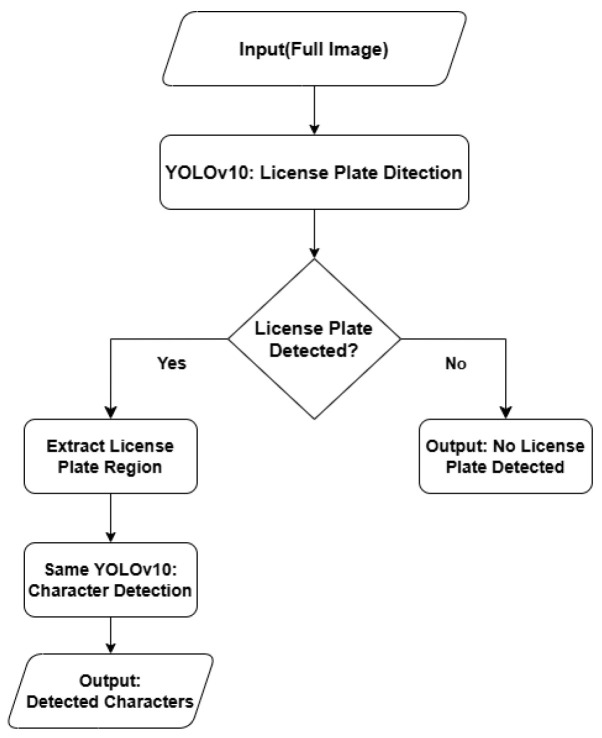
Detection workflow.

As shown in Figure 5, our detection process begins by inputting a full image into the YOLOv10 model, which first detects the license plate. If detected, the model extracts the plate region for character recognition using the same YOLOv10 model. If no plate is found, the system outputs “No License Plate Detected” and terminates. This two-stage method efficiently handles both plate localization and character recognition.

Algorithm 1 illustrates the full workflow in an algorithmic form.
**Algorithm 1** Weather-adaptive YOLOv10 for license plate detection and character recognition  1:**Input:** Image dataset D, License plate detection model $M_{YOLOv10}$, weather augmentation functions $A_{W}$  2:**Output:** Detected license plates and recognized characters  3:**procedure**TrainModel  4:      Initialize $M_{YOLOv10}$ with pre-trained COCO weights  5:      Apply data augmentations on D: flips, rotation, and color jittering  6:      Apply weather augmentation $A_{W}$ (rain, fog, noise)  7:      Train $M_{YOLOv10}$ on augmented dataset with batch size *B*, learning rate η, and epochs *E*  8:      Monitor precision, recall, and F1 score for training and validation  9:      Stop training if early stopping criteria are met10:**end procedure**11:**procedure**LicensePlateDetection12:      **Input:** Test image *I*13:      Pass *I* through $M_{YOLOv10}$ to detect license plates14:      **if** License plate detected **then**15:           Extract region of interest (ROI) containing the plate16:           Proceed to character recognition on extracted ROI17:      **else**18:           Output “No License Plate Detected”19:      **end if**20:**end procedure**21:**procedure**CharacterRecognition22:      Pass ROI through $M_{YOLOv10}$ to detect characters23:      Output detected characters24:**end procedure**25:**Evaluation:**26:Use metrics: Precision, Recall, F1 Score, and mAP for evaluation27:Evaluate

## 5. Results

In this section, we present and analyze the experimental results from training various YOLOv10 models with and without data augmentation. We compare six YOLOv10 variants: YOLOv10n, YOLOv10s, YOLOv10m, YOLOv10b, YOLOv10l, and YOLOv10x, using precision, recall, and F1 score as metrics [35]. The comparison is made across four conditions: without augmentation, with augmentation, weather without augmentation, and weather with augmentation. The results are summarized in the following tables.

### 5.1. Performance with Base Dataset

The performance analysis shows a clear improvement in the metrics as the models scale from YOLOv10n to YOLOv10m, with YOLOv10m achieving the best overall performance. It records the highest F1 score (91.69%), with balanced precision (94.24%) and recall (89.27%), as well as the highest mAP50 (94.75%) and competitive mAP50-95 (72.73%).

YOLOv10n, being the lightest model, has the lowest performance, with an F1 score of 84.92% and mAP50-95 of 69.25%, while YOLOv10s shows moderate improvement across all metrics. The larger models, including YOLOv10b, YOLOv10l, and YOLOv10x, maintain high performance, but with diminishing returns. YOLOv10b stands out with the highest mAP50-95 (72.92%), though YOLOv10x slightly underperforms in the F1 score (90.32%) and mAP metrics (Table 4).

### 5.2. Performance with General Augmentation

With general augmentation, all of the models demonstrate significant improvements in precision and F1 scores compared to the base dataset, with lower train losses indicating enhanced learning. YOLOv10m achieves the highest F1 score (93.34%) and precision (97.10%), reflecting the best balance between precision and recall (89.85%) among the models.

YOLOv10b and YOLOv10l follow closely, both achieving strong F1 scores (93.15%) and competitive mAP50-95 values (74.58% and 74.92%, respectively). YOLOv10x shows slightly reduced F1 performance (92.33%) but maintains high precision and mAP metrics. Meanwhile, YOLOv10n and YOLOv10s show marked improvements compared to their performance without augmentation, with YOLOv10s achieving an F1 score of 91.68% and mAP50-95 of 72.32% (Table 5).

### 5.3. Performance with Weather Augmentation

The application of weather augmentation further improves model performance, achieving results comparable to those with general augmentation while maintaining strong precision and recall metrics. YOLOv10m continues to excel, achieving an F1 score of 92.97%, with high precision (94.46%) and recall (91.53%), alongside the best mAP50 (94.90%) and competitive mAP50-95 (72.31%).

Other models, such as YOLOv10l and YOLOv10b, also perform well, with YOLOv10l achieving an F1 score of 92.57% and YOLOv10b showing balanced precision (91.94%) and recall (91.39%). YOLOv10x delivers consistent results, with an F1 score of 91.66% and the highest mAP50-95 (72.37%), emphasizing its reliability for detailed detections.

The lighter models, YOLOv10n and YOLOv10s, show improved performance compared to their base dataset results, with YOLOv10s reaching an F1 score of 92.07% and YOLOv10n achieving 91.51%, confirming the positive impact of weather augmentation. (Table 6).

### 5.4. Performance with General and Weather Augmentation

The combined effects of general and weather augmentation result in the best overall performance across all the models. YOLOv10x achieves the highest F1 score (94.36%) and precision (95.55%), demonstrating excellent generalization and robustness, with strong recall (93.20%) and the highest mAP50-95 (74.14%).

YOLOv10m and YOLOv10b also perform exceptionally well, with F1 scores of 93.38% and 93.74%, respectively. YOLOv10b shows a slight edge in mAP metrics (95.75% for mAP50 and 73.43% for mAP50-95), making it highly competitive for detailed detections. Similarly, YOLOv10l achieves a strong F1 score (93.72%) with the highest mAP50 (96.25%) and competitive mAP50-95 (74.05%).

The lighter models, YOLOv10n and YOLOv10s, also see notable improvements. YOLOv10s achieves an F1 score of 92.96% and a mAP50-95 of 72.15%, while YOLOv10n improves to an F1 score of 91.81%, reflecting the impact of combined augmentation (Table 7).

### 5.5. Performance Comparison

#### 5.5.1. Precision Comparison

**Figure 6:**
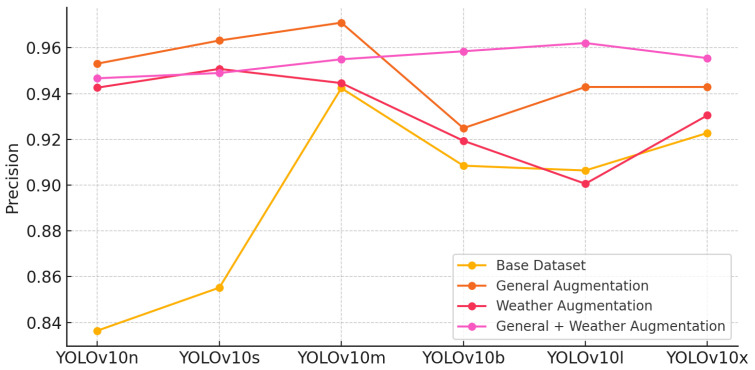
Precision comparison across YOLOv10 models and augmentation techniques.

The precision scores across the various YOLOv10 models show consistent improvement with the introduction of augmentation techniques. Models trained with general and weather augmentations achieved higher precision, indicating better accuracy in correctly identifying positive detections. Notably, YOLOv10m and YOLOv10l performed particularly well across all datasets, highlighting their robustness in diverse conditions. The highest precision was observed with general and weather augmentation, reflecting its effectiveness in enhancing detection accuracy (Figure 6).

#### 5.5.2. Recall Comparison

**Figure 7:**
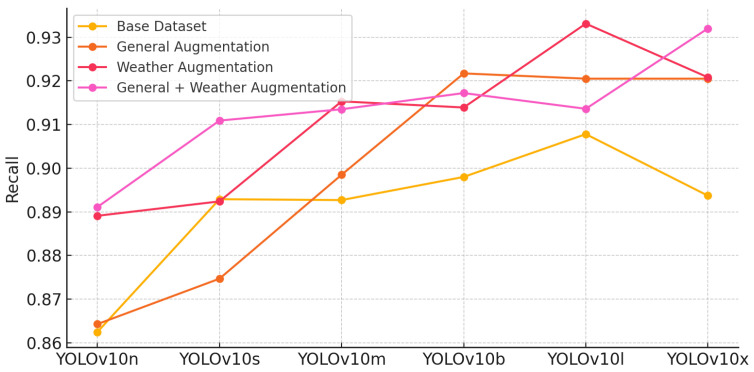
Precision comparison across YOLOv10 models and augmentation techniques.

The recall scores, representing the model’s ability to capture all relevant instances, also show improvement with augmentations. However, the increase in recall is not as prominent as precision, suggesting that while augmentation helps, some environmental factors still challenge complete detection. Models like YOLOv10x and YOLOv10l show more stability across datasets, especially with the combined augmentations, making them reliable choices in environments with varying weather conditions (Figure 7).

#### 5.5.3. F1 Comparison

**Figure 8:**
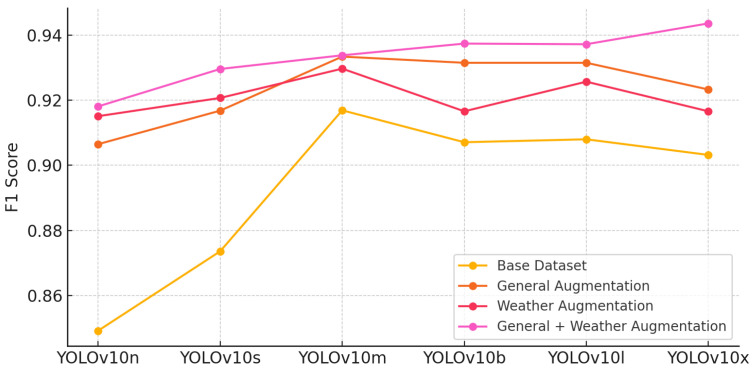
F1 comparison across YOLOv10 models and augmentation techniques.

The F1 score, which balances precision and recall, shows that models with both general and weather augmentations achieve the best overall performance. YOLOv10m and YOLOv10x maintain strong F1 scores, reflecting their ability to handle both false positives and false negatives effectively. The gradual improvement from base datasets to augmentation-enhanced datasets underscores the necessity of comprehensive augmentation techniques to ensure optimal performance across varying environmental conditions in ALPR tasks (Figure 8).

#### 5.5.4. mAP50 and mAP50-95 Comparison

The mAP50 and mAP50-95 metrics provide a comprehensive assessment of model localization and classification capabilities across varying IoU thresholds. The models trained with both general and weather augmentations exhibited the highest mAP50 values, particularly YOLOv10l and YOLOv10x, indicating superior performance in detecting objects with higher localization precision. Similarly, mAP50-95, which averages precision over a range of IoU thresholds, showed consistent improvements with augmentation, highlighting enhanced robustness across varying object sizes and positions.

**Figure 9:**
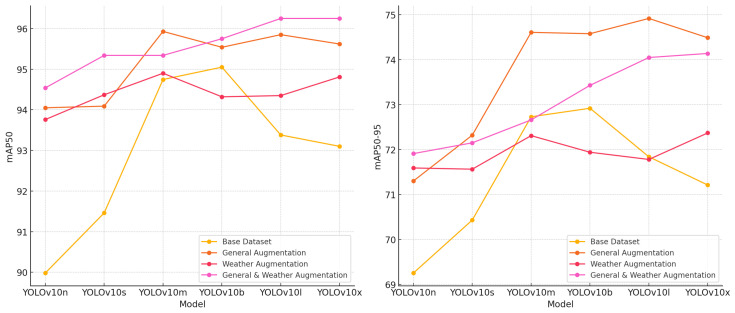
mAP50 and mAP50-95 comparisons across YOLOv10 models and augmentation techniques.

YOLOv10x, with general and weather augmentations, achieved the highest mAP50-95, emphasizing its ability to generalize across challenging scenarios and fine-grained detection thresholds. The progression from base datasets to augmentation-enhanced datasets underlines the critical role of diverse augmentation strategies in refining model adaptability and accuracy. These findings reinforce the importance of tailored augmentation techniques to achieve reliable performance in complex ALPR tasks, ensuring high accuracy in real-world applications (Figure 9).

### 5.6. Detection

**Figure 10:**
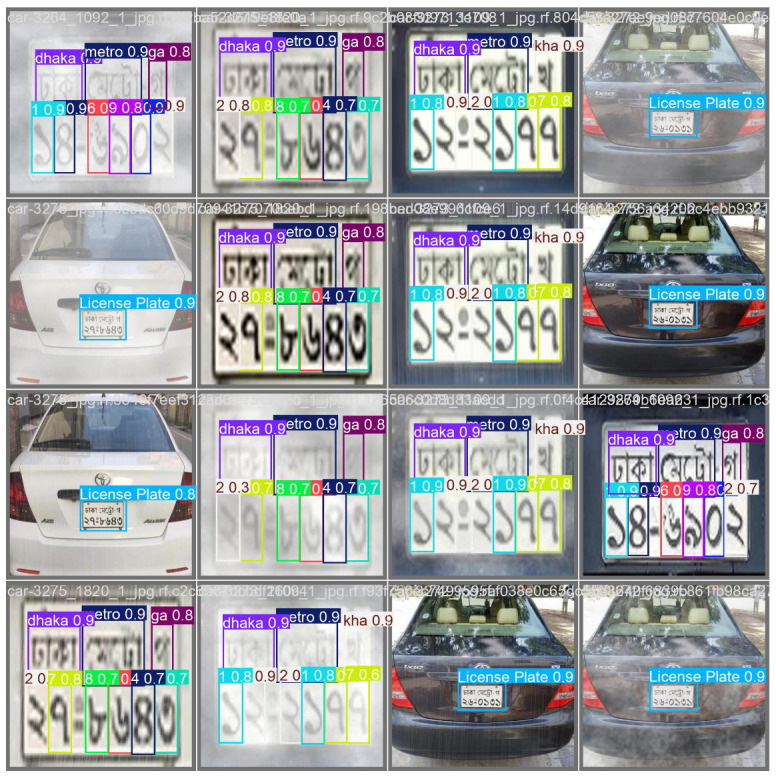
YOLO detection and recognition result grid under varied weather conditions.

Figure 10 shows the output of a YOLO10x model trained for both license plate detection and character recognition. It contains a series of photos of cars, each with a bounding box around the license plate labeled “License Plate” with a corresponding confidence score (e.g., 0.9 or 0.8). Alongside the license plate detection, individual characters on the plates are also identified, with each character enclosed in its own bounding box and assigned a confidence score, as well as labels like “dhaka”, “metro”, “ga”, and others. These labels likely correspond to different parts of the plate’s alphanumeric code, such as regional identifiers (e.g., “dhaka” or “metro”) and the characters or numbers that follow. The color-coded bounding boxes provide visual clarity on each segment recognized by the model, showcasing the effectiveness of the detection and recognition process. Some images are clearer than others, indicating variability in the quality of the predictions based on the input image.

In addition, the image highlights the model’s performance under varying weather conditions. For each license plate, the same image is shown under different environmental settings, such as foggy, overexposed, or clear conditions. Despite these changes in visibility, the model consistently detects the license plates and recognizes the individual characters, demonstrating its robustness across diverse scenarios. This visualization emphasizes not only the accuracy of detection and recognition but also the model’s resilience to challenging conditions such as poor lighting or fog, making this a comprehensive demonstration of its capabilities.

## 6. Conclusions

In conclusion, this paper introduces a weather-adaptive Convolutional Neural Network (CNN) framework based on the YOLOv10 model, designed to enhance the performance of Automatic License Plate Recognition (ALPR) systems under adverse weather conditions. By employing advanced data augmentation techniques, including weather-specific effects such as synthetic rain, fog, and noise, the proposed model significantly improves detection accuracy, even in challenging environments. The experimental results demonstrate marked improvements in precision, recall, F1, and mAP scores, validating the model’s robustness and adaptability across various environmental scenarios.

While this research addresses specific challenges encountered in regions like Bangladesh—such as diverse weather patterns, pollution, and dual-language license plates—the framework is inherently generalizable. The issues of adverse weather conditions and varying license plate designs are common in many parts of the world, and the proposed solutions can be adapted accordingly. The framework’s weather-adaptive capabilities ensure reliable performance, making it suitable for deployment in a wide range of geographical contexts beyond the initial case study. By focusing on the adaptability of data augmentation strategies and model architectures, this study underscores the scientific validity of the approach rather than limiting it to a specific regional application.

The successful application of this model indicates its potential for broader adoption. Future work could further refine these techniques and adapt them to other regions with unique weather conditions and license plate formats. Integrating the framework with real-time traffic management systems or broader intelligent transportation systems (ITS) would also enhance system scalability and operational efficiency. Exploring these avenues would provide valuable insights into the generalizability and adaptability of the model, reaffirming its relevance in diverse environmental and operational contexts.

Ultimately, the proposed framework presents a significant step forward in creating resilient, adaptable ALPR systems that are capable of maintaining high performance across a variety of challenging operational environments. By emphasizing transferability and general applicability, this research offers a promising solution for transportation management, law enforcement, and security in weather-affected regions worldwide.

## Figures and Tables

**Table 1:** Custom data augmentation techniques.

Augmentation Category	Type
Image flipping	Horizontal flip
	Vertical flip
Image rotation	±15°
	±90°
Color adjustment	Brightness adjustment
	Contrast adjustment
	Saturation adjustment
	Light exposure
Weather effects	Rain effect
	Fog effect
Noise injection	Gaussian noise
	Salt-and-pepper noise

**Table 2:** Performance metrics of YOLOv10 variants on the COCO dataset [33].

Model	Input Size	AP val (%)	FLOPs (G)	Latency (ms)
YOLOv10-N	640	38.5	6.7	1.84
YOLOv10-S	640	46.3	21.6	2.49
YOLOv10-M	640	51.1	59.1	4.74
YOLOv10-B	640	52.5	92.0	5.74
YOLOv10-L	640	53.2	120.3	7.28
YOLOv10-X	640	54.4	160.4	10.70

**Table 3:** Environmental setup for YOLOv10 training.

Parameter	Value
GPU	NVIDIA A100
GPU RAM	40 GB
CUDA version	12.3
Batch size	16
Number of epochs	50
Input image size	640 pixels
Learning rate	0.01
Momentum	0.937
Weight decay	0.0005

**Table 4:** Model performance with base dataset.

Model	Train Loss	Value Loss	Precision (%)	Recall (%)	F1 Score (%)	mAP50 (%)	mAP50-95 (%)
YOLOv10n	1.4510	1.8709	83.64	86.25	84.92	89.98	69.25
YOLOv10s	1.4927	2.1044	85.52	89.29	87.36	91.46	70.43
YOLOv10m	1.3089	2.0949	94.24	89.27	91.69	94.746	72.73
YOLOv10b	1.3034	2.1037	90.85	89.80	90.71	95.05	72.92
YOLOv10l	1.2993	2.1034	90.64	90.78	90.80	93.38	71.839
YOLOv10x	1.3035	2.1038	92.28	89.37	90.32	93.10	71.21

**Table 5:** Model performance with general augmentation.

Model	Train Loss	Value Loss	Precision (%)	Recall (%)	F1 Score (%)	mAP50 (%)	mAP50-95 (%)
YOLOv10n	1.4784	1.9268	95.31	86.43	90.65	94.05	71.30
YOLOv10s	1.2632	1.8800	96.32	87.47	91.68	94.09	72.32
YOLOv10m	1.1296	1.8614	97.10	89.85	93.34	95.93	74.61
YOLOv10b	1.1014	1.8834	92.49	92.17	93.15	95.54	74.58
YOLOv10l	1.0973	1.8708	94.29	92.05	93.15	95.85	74.92
YOLOv10x	1.0973	1.8708	94.29	92.05	92.33	95.62	74.49

**Table 6:** Model performance with weather augmentation.

Model	Train Loss	Value Loss	Precision (%)	Recall (%)	F1 Score (%)	mAP50 (%)	mAP50-95 (%)
YOLOv10n	1.2856	1.8913	94.26	88.91	91.51	93.76	0.7159
YOLOv10s	1.0304	2.0001	95.08	89.24	92.07	94.37	71563
YOLOv10m	0.9451	2.0500	94.46	91.53	92.97	94.90	72.31
YOLOv10b	0.9234	2.0471	91.94	91.39	91.66	94.32	71.94
YOLOv10l	0.9288	2.0803	90.06	93.31	92.57	94.35	71.78
YOLOv10x	0.9023	2.0960	93.05	92.08	91.66	94.81	72.37

**Table 7:** Model performance with general and weather augmentation.

Model	Train Loss	Value Loss	Precision (%)	Recall (%)	F1 Score (%)	mAP50 (%)	mAP50-95 (%)
YOLOv10n	1.3790	1.7225	94.67	89.11	91.81	94.54	71.91
YOLOv10s	1.0624	1.8298	94.90	91.09	92.96	95.34	72.15
YOLOv10m	0.9762	1.8942	95.50	91.35	93.38	95.34	72.66
YOLOv10b	1.3141	1.8303	95.85	91.72	93.74	95.75	73.43
YOLOv10l	1.0075	1.8742	96.21	91.36	93.72	96.25	74.05
YOLOv10x	1.0186	1.8388	95.55	93.20	94.36	96.25	74.14

## Data Availability

The base dataset used in this study is available at https://www.kaggle.com/datasets/syednahinhossain/bangladeshi-license-plate-recognition-dataset (accessed on 6 August 2024).
